# Developing Global Norms for Sharing Data and Results during Public Health Emergencies

**DOI:** 10.1371/journal.pmed.1001935

**Published:** 2016-01-05

**Authors:** Kayvon Modjarrad, Vasee S. Moorthy, Piers Millett, Pierre-Stéphane Gsell, Cathy Roth, Marie-Paule Kieny

**Affiliations:** World Health Organization, Geneva, Switzerland

## Abstract

Vasee Moorthy and colleagues describe the outcomes of a recent, WHO-led meeting on sharing research data and results during public health emergencies.

**Summary Points**

- Leading stakeholders from around the world convened at a WHO consultation in September 2015, where they affirmed that timely and transparent sharing of data and results during public health emergencies must become the global norm.
- Representatives from major biomedical journals who attended the meeting agreed that public disclosure of information of relevance to public health emergencies should not be delayed by publication timelines and that early disclosure should not and will not prejudice later journal publication.
- Researchers should be responsible for the accuracy of shared preliminary results, ensuring that they have been subjected to sufficient quality control before public dissemination.
- Opting in to data sharing should be the default practice, and the onus should be placed on data generators and stewards at the local, national, and international level to explain any decision to opt out from sharing data and results during public health emergencies.
- Incentives for sharing data should be created and tailored for each type of data generator and steward, while data management and analysis expertise is enhanced in under-resourced settings.

Among the core functions of the World Health Organization (WHO) are to provide “leadership on matters critical to health” and to shape “the research agenda and stimulate the generation, translation, and dissemination of valuable knowledge” in the interest of global public health [1]. These mandates have converged, of late, in the area of data sharing. In April 2015, the WHO adopted a position on the timely, public disclosure of clinical trial results [2], adding to other calls that nondisclosure of research data must no longer be tolerated. The global norm advocated by the WHO is to release key outcomes of all interventional clinical trials in an open-access, searchable database within 12 months of primary completion. The WHO has since called on sponsors, investigators, journals, and other bodies to do all in their power to implement this standard. Even as this position is becoming widely accepted, it has become clear that international standards are also needed for other types of data and results sharing, particularly in the context of public health emergencies.

When a new or re-emergent pathogen causes a major outbreak, rapid access to both raw and analysed data or other pertinent research findings becomes critical to developing a rapid and effective public health response. Without the timely exchange of information on clinical, epidemiologic, and molecular features of an infectious disease, informed decisions about appropriate responses cannot be made, particularly those that relate to fielding new interventions or adapting existing ones. Failure to share information in a timely manner can have disastrous public health consequences, leading to unnecessary suffering and death. The 2014–2015 Ebola epidemic in West Africa revealed both successful practices and important deficiencies within existing mechanisms for information sharing. For example, trials of two Ebola vaccine candidates (ChAd3-ZEBOV and rVSV-ZEBOV) benefited greatly from an open collaboration between investigators and institutions in Africa, Europe, and North America [3–6]. These teams, coordinated by the WHO, were able to generate and exchange critical data for the development of urgently needed, novel vaccines along faster timelines than have ever before been achieved. Similarly, some members of the genome sequencing community made viral sequence data publicly available within days of accessing samples [7], thus adhering to their profession’s long-established principles of rapid, public release of sequence data in any setting [8]. In contrast, the dissemination of surveillance data early in the epidemic was comparatively slow, and in some cases, the criteria for sharing were unclear.

In recognition of the need to streamline mechanisms of data dissemination—globally and in as close to real-time as possible—the WHO held a consultation in Geneva, Switzerland, on 1–2 September 2015 to advance the development of data sharing norms, specifically in the context of public health emergencies. Leading representatives from the scientific community, biomedical journals, industry, funding organizations, and government ministries from more than 20 low-, middle-, and high-income countries at all levels of research sector capacity convened to advance the development of core principles for sharing data swiftly and seamlessly. Meeting participants collectively identified several key obstacles to sharing data and results in times of acute public health need. Among these obstacles was the misperception that disclosure of major findings may negatively prejudice subsequent journal publication. Representatives from leading biomedical journals responded with an unequivocal assertion that public disclosure of information of relevance to public health emergencies should not be delayed by publication timelines and that early disclosure should not and will not prejudice journal publication of full scientific reports. Participants at the consultation agreed that sharing relevant information before publication should become the global norm during public health emergencies and that researchers should be responsible for ensuring that shared results—even when preliminary—have undergone some quality control and are, therefore, sufficiently accurate. These conditions will consequently enable an evidence-based dialogue with the media, affected communities, and other stakeholders.

Despite these reassurances from publishers, it was acknowledged that those generating data are often unable or unwilling to quickly transfer information beyond their research groups or collaborating networks because they either lack the technical capacity or harbour concerns that the data would be analysed and published without due recognition. There were also concerns that such data might lead to the development of products that source populations are unable to afford. The consensus solution was to enhance data management capacity and analytic expertise in under-resourced settings and to establish data transfer agreement templates now in order to set conditions in the future for the proper use of data and assignment of credit. Meeting participants also voiced concerns about the lack of disclosure and dissemination of negative results, as this may lead to unnecessary duplication of experiments, risks to human volunteers, and delays to effective product development. Thus, there was a call for investigators and sponsors to immediately disclose negative results generated prior to and during the Ebola outbreak. An intermediate step toward this end could entail a public log of all Ebola-related studies, including those with as-of-yet undisclosed results. An example of this practice is WHO’s malaria vaccine global portfolio table, which highlights unreported results (http://who.int/immunization/research/development/Rainbow_tables/en/).

Meeting participants also recognized that it is not enough for parties to simply agree, in principle, on sharing primary data, as the world must also commit to tackling the technical challenges of implementing data sharing agreements by simplifying and standardizing data capture procedures, assuring data quality, and harmonizing disparate data platforms. Broader issues must also be addressed, paramount of which is a gradual shift away from the culture of data ownership toward one of data stewardship. Although countries were recognized to be the key arbiters of the dissemination of data collected from their populations, it was also noted that data ultimately belong to the individuals from whom they are collected. Thus, in times of emergency, the onus should be placed upon the stewards of population- and individual-level data to justify if and why they are unwilling to share data for the good of public health. Although entities responsible for sharing data may raise valid concerns about the protection of privacy, it was also noted that, in the context of an emergency, there is as much or greater risk to both individual and public health posed by not sharing data. All those generating data during an emergency, therefore, have a moral obligation to share results as soon as interim findings are of sufficient quality. At the same time, incentives for sharing data—beyond moral responsibility—should be established and tailored for each sector, whether it is government, academia, or industry. The tension between the speed of data dissemination and its accuracy was also acknowledged. Thus, a mechanism for ensuring data quality must be embedded into any data sharing system, as major errors can degrade public confidence and have far-reaching impact.

Ultimately, preservation of global health requires prioritization of and support for international collaboration. These and other principles were affirmed at the consultation (**Table 1**) and codified into a consensus statement that was published on the WHO website immediately following the meeting (http://www.who.int/medicines/ebola-treatment/data-sharing_phe/en/). A more comprehensive set of principles and action items was made available in November 2015, including the consensus statement made by the editorial staff of journals that attended the meeting (http://www.who.int/medicines/ebola-treatment/blueprint_phe_data-share-results/en/). The success of prior initiatives to accelerate timelines for reporting clinical trial results has helped build momentum for a broader data sharing agenda. As the quick and transparent dissemination of information is the bedrock of good science and public health practice, it is important that the current trends in data sharing carry over to all matters of acute public health need. Such a global norm would advance the spirit of open collaboration, simplify current mechanisms of information sharing, and potentially save many lives in subsequent outbreaks.

**Table 1:** Issues and actions agreed on at the WHO consultation on data and results sharing during public health emergencies.

Issues	Actions
Perception that pre-publication disclosure of key results may prejudice journal publication.	A consensus statement from biomedical journals present at the meeting that pre-publication information sharing should become the norm during future emergencies, with parallel initiation of submission procedures to journals along longer timeframes.
Patchy public disclosure of genome sequence data.	A code of conduct, to be developed by the genome sequencing community and the WHO, for the public disclosure of genome sequence data in future public health emergencies.
Delays introduced by data use agreements.	Development of template data use agreements that outline governing principles for data sharing, benefits for those sharing data, responsibilities of those using data, and obligations to publicly disclose results of data analyses within specified timeframes.
Delays introduced by clinical trial agreements.	Development of template clinical trial and consortium agreements using United Nations jurisdiction to overcome contrasting national legal requirements.
Nondisclosure of epidemiologic data.	Opting in to rapid sharing should be considered the norm. The onus should be placed on data generators to explain any decision to opt out of data and results sharing.
Nondisclosure of clinical trial data.	Funders should change wording in agreements so that there is a requirement for expedited information sharing of quality-controlled interim results, as well as disclosure of final results when available.
	Call for public disclosure of existing Ebola results from animal models and clinical trials that are related to diagnostics, therapeutics, and/or prophylactics. In order to audit success in this area, a publicly available log of all conducted studies should be developed.
	Outside emergencies, 12 months is often considered an appropriate timeframe from study completion to public disclosure. In the emergency context, there was unanimity that 12 months should be greatly shortened from the time that interim results are available for public disclosure and that a specific expedited timeline commitment for results sharing should be made in protocols and analysis plans before trial commencement.
